# Supplementary material for: Chromothripsis during telomere crisis is independent of NHEJ, and consistent with a replicative origin
Source: Genome Res. 2019 May;29(5):737–49. doi: 10.1101/gr.240705.118 (PMC6499312; doi:10.1101/gr.240705.118)
Supplement: Supplemental Material [file supp_gr.240705.118_Supplemental_file_1.zip › contigs/annotated_contigs/DB54/contig.2.DB54_length_661_mean_cov_7.0015128593.docx]

**DB54_length_661_mean_cov_7.0015128593**

```
GCATTCTTCTTTTCTCAAAAGTGAACAAAAGAAAATTTCAGAAAATATAAAAATTCACTTAAGAAATGAATGGTTTACATTTGAAACCA
 >chr11:114812150-114812486 - E=2e-190
AATAAAGTTTGTCATGTGTTGAGTATCATTCTTGAGTGTGAAAAAAGGATACATTTGACCTAGATTGTAGGAACGTTGTCTATTGAGTG

GTGAAAGAATCATGATGTTGGCTTTATTGAGAAAGAAACATAATTCTAACTCATTTCTTATCTATGCAGTAAAACATATTTCTATAGAA

ATATTAATGAAAATATTAGTAACAATGGCCTAGGTTATGCTGAACTAACAAACCCAGCTCTCTTAATT|G|CACCATGCAGCCACTGCT
                                                                     >chr11:114769174-1
AGGGGATGGGGGAGGAGTGGAATCAGGGATTCAAGATTTTTTTTCTATCCTCTTCAGTGCCTGTTTTAGCTATATGAAGTTAAAACCAG
14769500 - E=2e-178
GTGCTGTGTGTGGTCACCTCATTTTTGGTTCTTATAAAGATTTTTGTTTGTTTGTTTTATTGTTTTTGGTATAGACAGTTGTTAAAGCA

CTGTCCTTAGAGCAGGGCCTGGGAAGATTGGTGGAGCCTTCTATTTGGCTATTTTGCTCCACCCCCCGTGTCAATGCAGTGACTTTTGA

AGATAACTGTCCTACTCTGCCCTCTAACATATGCCATTTG
```
